# Correction to Temperature and Bekenstein–Hawking Entropy of Kiselev Black Hole Surrounded by Quintessence

**DOI:** 10.3390/e27111135

**Published:** 2025-11-04

**Authors:** Cong Wang

**Affiliations:** College of Physics and Electronic Engineering, Qilu Normal University, Jinan 250300, China; wangcong@qlnu.edu.cn

**Keywords:** dark energy, quintessence, Kiselev black hole, Lorentz-breaking, Bekenstein–Hawking entropy

## Abstract

This paper studies a rotating Kiselev black hole surrounded by dark energy, whose spacetime metric is a solution to the Einstein field equations. Quintessence is a scalar field with negative pressure, related to the state parameter ω of the dark energy surrounding this black hole. Based on Lorentz-breaking, WKB approximation theory, and quantum tunneling radiation theory, we investigate the characteristic of quantum tunneling radition of spin-1/2 fermions and the result of the correction entropy in this special type of black hole. Additionally, we explore the significance of new expressions for physical quantities such as the Hawking temperature and Bekenstein–Hawking entropy of this black hole.

## 1. Introduction

Theoretical and observational studies in astronomy indicate that the universe is currently undergoing accelerated expansion. The dynamical mechanism behind this expansion has not yet been fully determined, leading to the proposal of various models of dark energy. These dark energy models can be broadly categorized into two types. The first type modifies the energy-momentum tensor on the right side of Einstein’s field equations, hoping to identify suitable new forms of matter that drive the accelerated expansion of the universe, such as the quintessence scalar field model. The second type aims to amend the mathematical theory of general relativity, which involves modifying the geometric part on the left side of Einstein’s field equations, encompassing various modified gravitational theories. Due to significant constraints on the adjustable parameters of these modified gravitational theories, the best model today remains the cosmological constant model, while the concept of new forms of matter is still unclear. The study of dark energy models continues to be a significant topic in contemporary astronomy. The accelerating expansion of the universe implies the contribution of matter with negative pressure to its evolution, which could be the cosmological constant or so-called quintessence matter. If quintessence matter exists throughout the universe, it can also be present around black holes. Therefore, astrophysical black holes are not isolated from matter. Up to now, it is not yet clear what kind of matter dominates the region around black holes. By considering quintessence matter as a source of energy-momentum, researchers have studied the rotating counterpart of the solution to the Einstein equations [1,2,3,4,5,6,7,8]. The rotational Kiselev black hole is surrounded by quintessence matter, and its quantum radiation characteristics represent a topic worthy of in-depth study. The quantum tunneling radiation of black holes can be divided into thermal and non-thermal radiation. Kraus et al. proposed a genuine tunneling radiation theory to investigate the Hawking thermal radiation of black holes [9,10,11,12,13,14,15]. Following this, researchers suggested using the semiclassical Hamilton–Jacobi method to study the quantum tunneling radiation of black holes [16,17,18]. Yang and Lin simplified the Dirac field equation into a matrix equation using semiclassical approximation theory and derived the Hamilton–Jacobi equation in curved spacetime by utilizing the commutation relations of Gamma matrices [19,20,21]. This method effectively simplifies the related studies of fermionic quantum tunneling radiation. In recent years, research on quantum gravity theory and string theory has indicated that Lorentz dispersion relations require modifications at the Planck scale in high-energy domains [22,23,24,25]. Lorentz-breaking can have significant effects on the dynamics of bosons and fermions in curved spacetime, making the study of modifications to curved spacetime and the quantum tunneling radiation characteristics of bosons and fermions a focal point of research [6,7,26,27,28,29,30,31]. However, in these significant studies, the influence of matter surrounding black holes has not been considered. Therefore, in this paper, we aim to research the impact of dark energy and Lorentz-breaking on the radiations of the Kiselev black hole surrounded by quintessence.

In the next two sections, we introduce the metric characteristics of the static, axially symmetric Kiselev black hole spacetime surrounded by quintessence matter, along with the modified form of the fermionic dynamics equation under Lorentz-breaking theory in this spacetime background. The third section discusses the radiation characteristics of the Kiselev black hole influenced by Lorentz-breaking and dark energy, as well as new expressions and implications for the modified Hawking temperature, Bekenstein–Hawking entropy, and other physical quantities. The final section of this paper discusses the research methods and conclusions derived from this study.

## 2. Quantum Tunneling Radiation Characteristics of Kiselev Black Hole Surrounded by Quintessence

Black holes in the universe do not exist in isolation. The existence of a black hole is related to the surrounding matter. Therefore, a real black hole spacetime metric should include factors concerning the surrounding matter. Kiselev studied the spacetime metric of a black hole surrounded by quintessence, which is a solution to the Einstein field equations. The Kiselev solution contemplates any type of energy-matter, once a state parameter has been established. The spacetime line element of a rotating Kiselev black hole is given by [6,7,8,9,10]

(1) $$\begin{array}{cc} ds^{2}= & -\left(1-\frac{2Mr+cr^{1-3\omega }}{\Sigma^{2}}\right)dt^{2}+\frac{\Sigma^{2}}{\Delta }dr^{2}+\Sigma^{2}d\theta^{2}+\sin^{2}\theta \left(r^{2}+a^{2}+a^{2}\sin^{2}\theta \frac{2Mr+cr^{1-3\omega }}{\Sigma^{2}}\right)d\phi^{2}\\ \; & -\frac{2a\sin^{2}\theta \left(2Mr+cr^{1-3\omega }\right)}{\Sigma^{2}}dtd\phi . \end{array}$$

where

(2) $$\begin{array}{cc} \;\Delta & =r^{2}-2Mr+a^{2}-cr^{1-3\omega }\\ \Sigma^{2} & =r^{2}+a^{2}\cos^{2}\theta \end{array}$$

Here, *M* is the mass of the black hole, and $a=\frac{J}{M}$ is the angular momentum per unit mass of this rotating black hole. *c* is the strength parameter. ω is the equation of state (EoS) parameter of the dark energy component. ω defines the EoS, p=ωρ, where *p* is the pressure, ρ is the energy density, and for dark energy, the range of ω is

(3) $$\begin{array}{c} -1<\omega <-\frac{1}{3}. \end{array}$$

It should be noted that the standard quintessence field is a perfect fluid in the cosmological sense, in the sense that no pressure anisotropy exists. However, in the black hole solution, different values of the quintessence parameter ω correspond to different kinds of energy-matter definable by the EoS. Specifically, dust corresponds to ω=0, radiation corresponds to $\omega =\frac{1}{3}$, the cosmological constant corresponds to ω=1, and the so-called $R_{h}$ universe corresponds to $\omega =-\frac{1}{3}$ [6,7,8,9,10]. Equation (1) describes a black hole wrapped in any kind of energy-matter definable by the EoS. In general, for dark energy, we would expect ω<0; quintessence, as said, would satisfy Equation (3). From Equations (1) and (2), it can be seen that the non-zero components of $g_{\mu \nu }$ are

(4) $$\begin{array}{cc} g_{tt} & =-\frac{1}{\Sigma^{2}}\left(\Sigma^{2}-2Mr-cr^{1-3\omega }\right)\\ g_{rr} & =\frac{\Sigma^{2}}{\Delta }\\ \;g_{\theta \theta } & =\Sigma^{2}\\ \;g_{\phi \phi } & =\sin^{2}\theta \left(r^{2}+a^{2}+a^{2}\sin^{2}\theta \frac{2Mr+cr^{1-3\omega }}{\Sigma^{2}}\right)\\ g_{t\phi } & =-\frac{a\sin^{2}\theta \left(2Mr+cr^{1-3\omega }\right)}{\Sigma^{2}} \end{array}$$

The determinant of the metric corresponding to $g_{\mu \nu }$ obtained from Equation (4) is

(5) $$\begin{array}{c} g=\left|g_{\mu \nu }\right|=-\Sigma^{4}\sin^{2}\theta =-{\left(r^{2}+a^{2}\cos^{2}\theta \right)}^{2}\sin^{2}\theta \end{array}$$

From Equations (4) and (5), as well as the algebraic cofactor $\Delta^{\mu \nu }$ of the metric $g_{\mu \nu }$, the non-zero components of the inverse metric tensor $g^{\mu \nu }$ can be calculated as $g^{\mu \nu }={\left(-1\right)}^{\mu +\nu }\frac{\Delta^{\mu \nu }}{g}$, and they are, respectively, as follows:

(6) $$\begin{array}{cc} g^{tt} & =-\frac{1}{\Delta }\left(r^{2}+a^{2}+a^{2}\sin^{2}\theta \frac{2Mr+cr^{1-3\omega }}{\Sigma^{2}}\right)\\ g^{rr} & =\frac{\Delta }{\Sigma^{2}}\\ g^{\theta \theta } & =\frac{1}{\Sigma^{2}}\\ g^{\phi \phi } & =\frac{\Delta -a^{2}\sin^{2}\theta }{\Delta \Sigma^{2}\sin^{2}\theta }\\ g^{t\phi } & =g^{\phi t}=-\frac{a}{\Delta \Sigma^{2}}\left(2Mr+cr^{1-3\omega }\right). \end{array}$$

In the four-dimensional curved spacetime described by Equation (1), a three-dimensional hypersurface can be represented by the equation f(t,r,θ,ϕ)=0, where *r*, θ, and ϕ are spherical coordinates. The normal vector is $n_{\mu }=\frac{𝜕f}{𝜕x^{\mu }}$, and the length of the normal vector is given by $n_{\mu }n^{\mu }=g^{\mu \nu }\frac{𝜕f}{𝜕x^{\mu }}\frac{𝜕f}{𝜕x^{\nu }}$. The hypersurface is a null surface if it satisfies the condition $n_{\mu }n^{\nu }=0$. The equation of the zero hypersurface that defines the event horizon of the black hole is as follows

(7) $$\begin{array}{c} g^{\mu \nu }\frac{𝜕f}{𝜕x^{\mu }}\frac{𝜕f}{𝜕x^{\nu }}=0 \end{array}$$

The equation for determining the event horizon of this black hole is $g^{rr}=0$, obtained by substituting Equation (6) into Equation (7), which gives

(8) $$\begin{array}{c} \Delta =r^{2}-2Mr+a^{2}-cr^{1-3\omega }=0. \end{array}$$

The horizons of this black hole depend on the equation of state ω and the strength parameter *c*. When −1<ω<−1/3, Equation (8) has three horizons corresponding to an event horizon $r_{+}$, a Cauchy horizon $r_{-}$, and a cosmological horizon. According to Equation (8), when ω≥−1/3, the cosmological horizon will disappear. We consider the case −1<ω<−1/3 and study the quantum tunneling radiation at the black hole’s $r_{H}$, where we only need to focus on the event horizon. From the equation, we can derive [7,8]

(9) $$\begin{array}{c} r_{+}=r_{H}=M+\sqrt{M^{2}-a^{2}}+\frac{c{\left(M+\sqrt{M^{2}-a^{2}}\right)}^{1-3\omega }}{2\sqrt{M^{2}-a^{2}}} \end{array}$$

Equations (1)–(9) describe the spacetime characteristics of the Kiselev black hole surrounded by quintessence. In the curved spacetime of this black hole, the dynamics of bosons and fermions have new expressions after Lorentz-breaking theoretical corrections. Therefore, physical quantities such as the quantum tunneling radiation rate at the event horizon $r_{H}$ of this black hole will have new results. The following content will present an expression for this topic, which has not been deeply studied. With the expression for $r_{H}$, we can study the quantum tunneling radiation characteristics of bosons or fermions at the event horizon of this black hole, as well as related topics. Considering spin-1/2 fermions, which are Dirac particles, and taking into account the Lorentz-breaking correction effects. Nascimento et al. studied the action of the spinor field $\bar{S}$ in flat spacetime and the application of Lorentz-breaking theories, proposing the higher-derivative extension of spinor QED in 4D as follows [26,27]: $\bar{S}=\int d^{4}x\bar{\varphi }\left[i\gamma^{m}D_{m}\left(1-\alpha \frac{\gamma^{m}\gamma^{n}D_{m}D_{n}}{m^{2}}\right)+b\gamma^{5}+\xi {\left(bD\right)}^{2}-m\right]\varphi$, where $D_{m}=\alpha m-ieA_{m}$ is the usual gauge covariant derivative. $\bar{\varphi }$ is the conjugate of φ. α,ξ,b are the coefficients of the CFJ modification term, the aether-like modification term, and the chiral modification term, respectively. The action of the spinor field in the Kiselev black hole spacetime, as expressed by Equations (1) and (2), can be modified to [31]

(10) $$\begin{array}{cc} S_{f}= & \int d^{4}x\sqrt{-g}\tilde{\psi }\left\{i\gamma^{\mu }\left({𝜕}_{\mu }+\frac{ie}{\hbar }A_{\mu }+i\Omega_{\mu }\right)\left[1-\frac{a_{0}\hbar^{2}}{m^{2}}\gamma^{\mu }\gamma^{\nu }\left({𝜕}_{\mu }+\frac{ie}{\hbar }A_{\mu }+i\Omega_{\mu }\right)\left({𝜕}_{\nu }+\frac{ie}{\hbar }A_{\nu }+i\Omega_{\nu }\right)\right]\right.\\ \; & \left.+\frac{\lambda \hbar }{m}u^{\mu }u^{\nu }\left({𝜕}_{\mu }+\frac{ie}{\hbar }A_{\mu }+i\Omega_{\mu }\right)\left({𝜕}_{\nu }+\frac{ie}{\hbar }A_{\nu }+i\Omega_{\nu }\right)+\frac{b_{0}\gamma^{5}}{m\hbar }-\frac{m}{\hbar }\right\}\psi =\int d^{4}x\sqrt{-g}L_{f} \end{array}$$

According to the variational principle, we can obtain

(11) $$\begin{array}{c} \delta S_{f}=\int d^{4}x\sqrt{-g}\delta L_{f}=0 \end{array}$$

From Equations (10) and (11), we obtain the modified spin field equation as follows

(12) $$\begin{array}{cc} \; & \left\{i\gamma^{\mu }\left({𝜕}_{\mu }+\frac{ie}{\hbar }A_{\mu }+i\Omega_{\mu }\right)\left[1-\frac{a_{0}\hbar^{2}}{m^{2}}\gamma^{\mu }\gamma^{\nu }\left({𝜕}_{\mu }+\frac{ie}{\hbar }A_{\mu }+i\Omega_{\mu }\right)\left({𝜕}_{\nu }+\frac{ie}{\hbar }A_{\nu }+i\Omega_{\nu }\right)\right]\right.\\ \; & \left.+\frac{\lambda \hbar }{m}u^{\mu }u^{\nu }\left({𝜕}_{\mu }+\frac{ie}{\hbar }A_{\mu }+i\Omega_{\mu }\right)\left({𝜕}_{\nu }+\frac{ie}{\hbar }A_{\nu }+i\Omega_{\nu }\right)+\frac{b_{0}\gamma^{5}}{m\hbar }-\frac{m}{\hbar }\right\}\psi =0 \end{array}$$

In Equation (10), $\tilde{\psi }$ is the conjugate of ψ. In Equations (10) and (12), $a_{0}$, $b_{0}$ and λ are coefficients of small correction terms. Here, $a_{0}$ corresponds to the CFJ term, $b_{0}$ corresponds to the Chiral correction term, and λ corresponds to the aether-like correction term [26,27,32,33,34]. According to the WKB approximation theory and black hole quantum tunneling radiation theory, spin-1/2 fermions, also known as Dirac particles, are considered. For Dirac particles, the wave function in Equation (12) can be expressed as

(13) ψ=ABeiSℏ

Here, *A* and *B* are the matrix elements of the 2×1 column matrices. Substituting Equation (13) into Equation (12) and multiplying both sides of Equation (12) by *ℏ*, while omitting the spin connection term $\hbar \Omega_{\mu }$ that contains *ℏ*, we can obtain the equation for the Dirac particle action *S* as follows:

(14) −γμ𝜕μS+1−a0m2γμγν𝜕μS+eAμ𝜕νS+eAν−λmuμuν𝜕μS+eAμ𝜕νS+eAν+b0γ5m−mAB=0

This is a matrix equation, which is actually an eigen matrix equation. The condition of non-trivial solution for this equation to is that the determinant of the corresponding matrix is zero, i.e.,

(15) $$\begin{array}{c} \gamma^{\mu }{𝜕}_{\mu }S+\left[1-\frac{a_{0}}{m^{2}}\gamma^{\mu }\gamma^{\nu }\left({𝜕}_{\mu }S+eA_{\mu }\right)\left({𝜕}_{\nu }S+eA_{\nu }\right)\right]\left[-\frac{\lambda }{m}u^{\mu }u^{\nu }\left({𝜕}_{\mu }S+eA_{\mu }\right)\left({𝜕}_{\nu }S+eA_{\nu }\right)+\frac{b_{0}\gamma^{5}}{m}-m\right]=0. \end{array}$$

The gamma matrices in Equation (15) are constrained by the following conditions:

(16) $$\begin{array}{c} \gamma^{\mu }\gamma^{\nu }+\gamma^{\nu }\gamma^{\mu }=2g^{\mu \nu }I \end{array}$$

(17) $$\begin{array}{c} \gamma^{\mu }\gamma^{5}+\gamma^{5}\gamma^{\mu }=0 \end{array}$$

Thus, it is easy to obtain from Equations (15) and (16) that

(18) $$\begin{array}{c} \left[g^{\mu \nu }\left(1-2a_{0}\right)+2\lambda u^{\mu }u^{\nu }\right]\left({𝜕}_{\mu }S+eA_{\mu }\right)\left({𝜕}_{\nu }S+eA_{\nu }\right)+m^{2}-2b_{0}\gamma_{0}^{5}=0. \end{array}$$

This is the spin field equation for spin-1/2 fermions. In this equation, $g^{\mu \nu }$, $u^{\mu }$ and $\gamma_{0}^{5}$ need to be determined based on the specific characteristics of the curved spacetime. The conditions $u^{\mu }u_{\mu }=\mathrm{const}.$, and $\gamma^{5}$ and $\gamma^{\mu }$ must satisfy Equations (16) and (17). To solve Equation (18), the components of the aether-like field vector $u^{\mu }$ are chosen as follows:

(19) $$\begin{array}{c} \begin{array}{cc} u_{t}=\frac{c_{t}}{\sqrt{g^{tt}}}, & u_{t}u^{t}=u_{t}u_{t}g^{tt}=c_{t}^{2}\\ u_{r}=\frac{c_{r}}{\sqrt{g^{rr}}}, & u_{r}u^{r}=u_{r}u_{r}g^{rr}=c_{r}^{2}\\ u_{\theta }=\frac{c_{\theta }}{\sqrt{g^{\theta \theta }}}, & u_{\theta }u^{\theta }=u_{\theta }u_{\theta }g^{\theta \theta }=c_{\theta }^{2}\\ u_{\phi }=\frac{c_{\phi }}{\sqrt{g^{\phi \phi }}}, & u_{\phi }u^{\phi }=u_{\phi }u_{\phi }g^{\phi \phi }=c_{\phi }^{2} \end{array} \end{array}$$

Clearly, $u_{\mu }u^{\mu }=u_{\mu }u_{\nu }g^{\mu \nu }=2c_{t}^{2}+c_{r}^{2}+c_{\theta }^{2}+2c_{\phi }^{2}=\alpha_{0}^{2}\left(\mathrm{const}\right)$. To facilitate solving Equation (18), we choose the four components of $u_{\mu }$ as shown in Equation (19). In order to determine the specific forms of $\gamma^{5}$ and $\gamma_{0}^{5}$, we choose the four components of the gamma matrices in the Kiselev spacetime as follows:

(20) $$\begin{array}{cc} \gamma^{t} & =\sqrt{g^{tt}}\left(\begin{array}{cc} I & 0\\ 0 & -I \end{array}\right)\\ \gamma^{r} & =\sqrt{g^{rr}}\left(\begin{array}{cc} 0 & \sigma^{1}\\ \sigma^{1} & 0 \end{array}\right)\\ \gamma^{\theta } & =\sqrt{g^{\theta \theta }}\left(\begin{array}{cc} 0 & \sigma^{2}\\ \sigma^{2} & 0 \end{array}\right)\\ \gamma^{\phi } & =\frac{g^{t\phi }}{\sqrt{g^{tt}}}\left(\begin{array}{cc} I & 0\\ 0 & -I \end{array}\right)+\sqrt{\frac{g^{tt}g^{\phi \phi }-{\left(g^{t\phi }\right)}^{2}}{g^{tt}}}\left(\begin{array}{cc} 0 & \sigma^{3}\\ \sigma^{3} & 0 \end{array}\right) \end{array}$$

where I is the identity matrix, and $\sigma^{i}\left(i=1,2,3\right)$ are the Pauli matrices, i.e.,

(21) $$\begin{array}{c} \sigma^{1}=\left(\begin{array}{cc} 0 & 1\\ 1 & 0 \end{array}\right),\sigma^{2}=\left(\begin{array}{cc} 0 & -i\\ i & 0 \end{array}\right),\sigma^{3}=\left(\begin{array}{cc} 1 & 0\\ 0 & 1 \end{array}\right) \end{array}$$

According to the characteristics of the Kiselev spacetime metric, $\gamma^{5}$ is chosen as follows:

(22) $$\begin{array}{cc} \; & \gamma^{5}=\frac{1}{r^{2}\sqrt{-\frac{g}{g_{rr}g_{\theta \theta }}}}\gamma^{t}\gamma^{r}\gamma^{\theta }\left[\gamma^{\phi }-\frac{g^{t\phi }}{\sqrt{g^{tt}}}\left(\begin{array}{cc} I & 0\\ 0 & I \end{array}\right)\right]\\ \; & \;=\frac{\Delta }{i\Sigma^{2}r^{2}}\left(\begin{array}{cc} I & 0\\ 0 & -I \end{array}\right)\left(\begin{array}{cc} 0 & \sigma^{1}\\ \sigma^{1} & 0 \end{array}\right)\left(\begin{array}{cc} 0 & \sigma^{2}\\ \sigma^{2} & 0 \end{array}\right)\left(\begin{array}{cc} 0 & \sigma^{3}\\ \sigma^{3} & 0 \end{array}\right)=\gamma_{0}^{5}\left(\begin{array}{cc} I & 0\\ 0 & I \end{array}\right) \end{array}$$

where

(23) $$\begin{array}{c} \gamma_{0}^{5}=\frac{\Delta }{r^{2}\Sigma^{2}}=\frac{r^{2}-2Mr+a^{2}-cr^{1-3\omega }}{r^{2}\left(r^{2}+a^{2}\cos^{2}\theta \right)} \end{array}$$

Substituting Equations (16), (19) and (21) into the field equation Equation (18), we obtain the dynamical equation for spin-1/2 fermions in the Kiselev spacetime as

(24) $$\begin{array}{cc} \; & -\frac{1}{\Delta }\left(1-2a_{0}+2\lambda c_{t}^{2}\right)\left[\Sigma^{2}\left(r^{2}+a^{2}\right)+a^{2}\left(2Mr+cr^{1-3\omega }\right)-a^{2}\cos^{2}\theta \left(2Mr+cr^{1-3\omega }\right)\right]{\left(\frac{𝜕S}{𝜕t}\right)}^{2}\\ \; & +\Delta \left(1-2a_{0}+2\lambda c_{r}^{2}\right){\left(\frac{𝜕S}{𝜕r}\right)}^{2}-\frac{2}{\Delta }\left(1-2a_{0}+2\lambda c_{\phi }^{2}\right)a\Sigma^{2}\left(2Mr+cr^{1-3\omega }\right)\left(\frac{𝜕S}{𝜕t}\right)\left(\frac{𝜕S}{𝜕\phi }\right)\\ \; & +\frac{1}{\Delta }\left(1-2a_{0}+2\lambda c_{\phi }^{2}\right)\left(\frac{\Delta }{\sin^{2}\theta }-a^{2}\right){\left(\frac{𝜕S}{𝜕\phi }\right)}^{2}+\Sigma^{2}\left[m^{2}-2b_{0}\gamma_{0}^{5}+g_{\theta \theta }\left(1-2a_{0}+2\lambda c_{\theta }^{2}\right)\right]{\left(\frac{𝜕S}{𝜕\theta }\right)}^{2}\\ \; & +\Sigma^{2}\left(m^{2}-2b_{0}r_{0}^{5}\right)+\lambda u^{r}\left(u^{t}\frac{𝜕S}{𝜕r}\frac{𝜕S}{𝜕t}+u^{\theta }\frac{𝜕S}{𝜕r}\frac{𝜕S}{𝜕\theta }+u^{\phi }\frac{𝜕S}{𝜕r}\frac{𝜕S}{𝜕\phi }\right)+4\lambda u^{\theta }{\left(u^{t}\frac{𝜕S}{𝜕\theta }\frac{𝜕S}{𝜕t}+u^{\phi }\frac{𝜕S}{𝜕\theta }\frac{𝜕S}{𝜕\phi }\right)}^{2}=0 \end{array}$$

where $\Sigma^{2}=r^{2}+a^{2}\cos^{2}\theta =r^{2}+a^{2}-a^{2}\sin^{2}\theta$. The constant introduced when separating variables in Equation (24) is denoted as $d_{0}$. Multiplying both sides of this equation by Δ, and noting that ${\Delta |}_{r\to r_{H}}=0$, the radial motion equation for spin-1/2 particle, derived from Equation (24) at the black hole horizon, is given by

(25) $$\begin{array}{cc} \; & -\left(1-2a_{0}+2\lambda c_{t}^{2}\right)\left[\left(r_{H}^{2}+a^{2}\right)r_{H}^{2}+a^{2}\left(2Mr_{H}+cr_{H}^{1-3\omega }\right)\right]{\left(\frac{𝜕S}{𝜕t}\right)}^{2}\\ \; & +\left(1-2a_{0}+2\lambda c_{r}^{2}\right){\left(\Delta \frac{𝜕S}{𝜕r}\right)}_{r\to r_{H}}^{2}+2\left(1-2a_{0}+2\lambda c_{\phi }^{2}\right)a\left(2Mr_{H}+cr_{H}^{1-3\omega }\right)\frac{𝜕S}{𝜕t}\frac{𝜕S}{𝜕\phi }\\ \; & -\left(1-2a_{0}+2\lambda c_{\phi }^{2}\right)a^{2}{\left(\frac{𝜕S}{𝜕\phi }\right)}^{2}+{\left[{\Delta |}_{r\to r_{H}}\left(m^{2}-2b_{0}\gamma_{0}^{5}\right)r-\Delta d_{0}\right]}_{r\to r_{H}}=0 \end{array}$$

The Kiselev spacetime possesses a Killing vector $\frac{𝜕}{𝜕\phi }$, so $\frac{𝜕S}{𝜕\phi }=n\left(\mathrm{const}.\right)$ the particle action *S* in Equation (25) can be separated, i.e.,

(26) $$\begin{array}{c} S=-Et+R(r)+\Theta (\theta )+n\phi \end{array}$$

After substituting Equation (26) into Equation (25), the following is obtained:

(27) $$\begin{array}{cc} {\left(\Delta \frac{dR}{dr}\right)}_{r\to r_{H}}^{2} & =\frac{1-2a_{0}+2\lambda c_{t}^{2}}{1-2a_{0}+2\lambda c_{r}^{2}}\left\{{\left[E\left(r_{H}^{2}+a^{2}\right)\right]}^{2}-2anE\left(2Mr_{H}+cr_{H}^{1-3\omega }\right)+a^{2}n^{2}\right\}\\ \; & =\frac{1-2a_{0}+2\lambda c_{t}^{2}}{1-2a_{0}+2\lambda c_{r}^{2}}{\left(r_{H}^{2}+a^{2}\right)}^{2}{\left(E-E_{0}\right)}^{2} \end{array}$$

Got Equation (27) before we used $u^{t}u^{\phi }=g^{t\phi }u_{\phi }u^{\phi }=g^{t\phi }c_{\phi }^{2}$ and $u^{t}u^{\phi }=u^{t}u_{t}g^{t\phi }=g^{t\phi }c_{t}^{2}$. From Equation (27), we obtain

(28) $$\begin{array}{cc} E_{0} & =\frac{an}{r_{H}^{2}+a^{2}} \end{array}$$

(29) $$\begin{array}{cc} {\left.\frac{dR}{dr}\right|}_{r\to r_{H}} & =\pm \frac{1}{{\left.\Delta \right|}_{r\to r_{H}}}{\left(\frac{1-2a_{0}+2\lambda c_{t}^{2}}{1-2a_{0}+2\lambda c_{r}^{2}}\right)}^{1/2}\left(r_{H}^{2}+a^{2}\right)\left(E-E_{0}\right). \end{array}$$

The application of the residue theorem to integrate both sides of Equation (28) yields

(30) $$\begin{array}{c} R_{\pm }=\pm \frac{i\pi \left(r_{H}^{2}+a^{2}\right)}{\Delta^{\prime }\left(r_{H}\right)}{\left(\frac{1-2a_{0}+2\lambda c_{t}^{2}}{1-2a_{0}+2\lambda c_{r}^{2}}\right)}^{1/2}\left(E-E_{0}\right), \end{array}$$

where

(31) $$\begin{array}{c} \Delta^{\prime }\left(r_{H}\right)=2r_{H}-2M-c\left(1-3\omega \right)r_{H}^{-3\omega }. \end{array}$$

Based on the WKB approximation and the quantum tunneling radiation theory, the quantum tunneling rate of the semiclassical at the event horizon $r_{H}$ of the Kiselev black hole is given by

(32) Γ∼exp−2ImS±=exp−2ImR±=exp−E−E0TH,

where

(33) $$\begin{array}{c} T_{H}=\frac{2r_{H}-2M+c\left(1-3\omega \right)r_{H}^{1-3\omega }}{4\pi \left(r_{H}^{2}+a^{2}\right)}{\left(\frac{1-2a_{0}+2\lambda c_{r}^{2}}{1-2a_{0}+2\lambda c_{t}^{2}}\right)}^{1/2}. \end{array}$$

To elucidate the relationship between the corrected entropy and the *ℏ*-perturbation, Equations (29) and (30) can be, respectively, rewritten in the following forms:

(34) $$\begin{array}{c} \frac{dR_{0}^{\pm }}{dr}{|}_{r\to r_{H}}=\pm \frac{1}{{\Delta |}_{r\to r_{H}}}{\left(\frac{1-2a_{0}+2\lambda c_{t}^{2}}{1-2a_{0}+2\lambda c_{r}^{2}}\right)}^{1/2}\left(r_{H}^{2}+a^{2}\right)\left(E-E_{0}\right) \end{array}$$

(35) $$\begin{array}{c} R_{0}^{\pm }=\pm \frac{i\pi \left(r_{H}^{2}+a^{2}\right)}{\Delta^{\prime }\left(r_{H}\right)}{\left(\frac{1-2a_{0}+2\lambda c_{t}^{2}}{1-2a_{0}+2\lambda c_{r}^{2}}\right)}^{1/2}\left(E-E_{0}\right) \end{array}$$

Let

(36) $$\begin{array}{c} E=E_{0}^{\prime }+\sum_{i=1}\hbar^{i}E_{i} \end{array}$$

(37) $$\begin{array}{c} R^{\pm }=R_{0}^{\pm }+\sum_{i=1}\hbar^{i}R_{i}^{\pm } \end{array}$$

where $E_{0}^{\prime }=E-E_{0}$. From Equation (29) to Equation (37), the following relationship can be derived [35,36]

(38) $$\begin{array}{c} \frac{dR_{1}^{\pm }}{dr}{|}_{r\to r_{H}}=\pm \frac{E_{1}\left(r_{H}^{2}+a^{2}\right)}{{\Delta |}_{r_{1}\to r_{H}}}{\left(\frac{1-2a_{0}+2\lambda c_{t}^{2}}{1-2a_{0}+2\lambda c_{r}^{2}}\right)}^{1/2} \end{array}$$

(39) $$\begin{array}{cc} \; & \frac{dR_{2}^{\pm }}{dr}{|}_{r\to r_{H}}=\pm \frac{E_{2}\left(r_{H}^{2}+a^{2}\right)}{{\Delta |}_{r_{1}\to r_{H}}}{\left(\frac{1-2a_{0}+2\lambda c_{t}^{2}}{1-2a_{0}+2\lambda c_{r}^{2}}\right)}^{1/2}\\ \; & \vdots \end{array}$$

From Equations (9), (38) and (39), it is clear that an inherent relationship exists between $R_{1}^{\pm }$, $R_{2}^{\pm }$, $R_{3}^{\pm }\cdots$ and $R_{0}^{\pm }$. Defining $\frac{R_{i}^{\pm }}{R_{i+1}^{\pm }}=\alpha_{i}^{\prime }$, Given $E_{i}/E_{i-1}=\beta_{i}$, Equations (36) and (37) can be rewritten as $E=E_{0}^{\prime }+\sum_{i=1}\hbar^{i}\beta_{i}E_{0}^{\prime }$, $R^{\pm }=R_{0}^{\pm }+\sum_{i=1}\hbar^{i}\alpha_{i}^{\prime }R_{0}^{\pm }$. where $\alpha_{i}^{\prime }$ can be expressed as $\alpha_{i}/S_{\mathrm{BH}}$. $T_{H}^{\prime }$ denotes the Hawking temperature at the event horizon of this black hole, which includes terms with *ℏ* corrections. According to Equation (37), the quantum tunneling rate of this black hole can be rewritten as

(40) $$\begin{array}{cc} \Gamma^{\prime }∼\exp\left(-2I_{m}R_{i}^{\pm }\right) & =\exp\left[-4\pi \frac{\left(r_{H}^{2}+a^{2}\right)E_{0}^{\prime }}{\Delta^{\prime }\left(r_{H}\right)}{\left(\frac{1-2a_{0}+2\lambda c_{t}^{2}}{1-2a_{0}+2\lambda c_{r}^{2}}\right)}^{1/2}\left(1+\sum_{i=1}\hbar^{i}\alpha_{i}^{\prime }\right)\right]\\ \; & =\exp\left(-\frac{E_{0}^{\prime }}{T_{H}^{\prime }}\right) \end{array}$$

where

(41) $$\begin{array}{c} T_{H}^{\prime }=\frac{\Delta^{\prime }\left(r_{H}\right)}{4\pi \left(r_{H}^{2}+a^{2}\right)}{\left(\frac{1-2a_{0}+2\lambda c_{t}^{2}}{1-2a_{0}+2\lambda c_{r}^{2}}\right)}^{-1/2}{\left(1+\sum_{i=1}\hbar^{i}\alpha_{i}^{\prime }\right)}^{-1}=T_{H}{\left(1+\sum_{i=1}\hbar^{i}\alpha_{i}/S_{BH}\right)}^{-1} \end{array}$$

For this black hole, the corrected first law of thermodynamics is formulated as follows:

(42) $$\begin{array}{c} dM=T_{H}^{\prime }dS_{BH}^{\prime }+\Omega dJ-\rho dV_{d} \end{array}$$

Thus,

(43) $$\begin{array}{cc} S_{BH}^{\prime } & =\int \frac{dM-\Omega dJ-\rho dV_{d}}{T_{H}^{\prime }}\\ \; & =S_{BH}+\hbar \alpha_{1}\ln S_{BH}+\cdots \end{array}$$

where

(44) $$\begin{array}{cc} S_{BH} & =\int \frac{dM-\Omega dJ-edV_{d}}{T_{H}}=\int \frac{dM-\Omega dJ-edV_{d}}{T_{0}}{\left(\frac{1-2a_{0}+2\lambda c_{t}^{2}}{1-2a_{0}+2\lambda c_{r}^{2}}\right)}^{1/2}\\ \; & =S_{bh}{\left(\frac{1-2a_{0}+2\lambda c_{t}^{2}}{1-2a_{0}+2\lambda c_{r}^{2}}\right)}^{1/2} \end{array}$$

where $T_{0}=\frac{\Delta^{\prime }\left(r_{H}\right)}{4\pi (r_{H}^{2}+c_{r}^{2})}$ is the original temperature of the black hole, and $S_{bh}$ is the original Bekensein–Hawking entropy. Equation (43) is the logarithmic-corrected version of the Bekenstein–Hawking entropy of this black hole. The logarithmic-corrected version of the Bekenstein–Hawking entropy is approved by various quantum efforts such as conical singularity and entanglement entropy, Euclidean action method, quantum geometry, Candy formula, conformal anomaly, Noether charge, and non-locality of quantum gravity [35,36]. According to the new expression for the Bekenstein–Hawking entropy of the Kiselev black hole and the second law of black hole thermodynamics, the black hole entropy never decreases in the clockwise direction, i.e.,

(45) $$\begin{array}{c} \Delta S_{BH}^{\prime }\geq 0 \end{array}$$

Therefore, the expression for the black hole’s quantum tunneling radiation rate in Equation (40) can be rewritten as

(46) $$\begin{array}{c} \Gamma =\exp\left(\Delta S_{BH}^{\prime }\right). \end{array}$$

The area of the black hole event horizon is $A_{k}$. Due to

(47) $$\begin{array}{c} dH^{2}=\Sigma^{2}d\theta^{2}+\sin^{2}\theta \left(r_{H}^{2}+a^{2}+a^{2}\sin^{2}\theta \frac{2Mr_{H}+cr_{H}^{1-3\omega }}{\Sigma^{2}}\right)d\phi^{2}, \end{array}$$

the metric determinant corresponding to Equation (47) is

(48) $$\begin{array}{c} g_{H}=\sin^{2}\theta \left(r_{H}^{2}+a^{2}\right)\left(r_{H}^{2}+a^{2}\cos^{2}\theta \right)+a^{2}\sin^{2}\theta \left(r_{H}^{2}+a^{2}\right)=\sin^{2}\theta {\left(r_{H}^{2}+a^{2}\right)}^{2} \end{array}$$

where $r_{H}$ is related to *c* and ω.

(49) $$\begin{array}{c} A_{k}=\int \sqrt{g_{H}}d\theta d\phi =4\pi \left(r_{H}^{2}+a^{2}\right)=4\pi \left(2Mr_{H}+cr_{H}^{1-3\omega }\right) \end{array}$$

so,

(50) $$\begin{array}{c} S_{bh}=A_{k}/4 \end{array}$$

Thus, the semiclassical Bekenstein–Hawking area law is given by $S_{bh}=\frac{A_{k}}{4}=\pi {\left(2Mr_{H}+cr_{H}^{1-3\omega }\right)}^{2}$ represents the uncorrected original Bekenstein–Hawking entropy of the black hole and $A_{k}$ is the area of the event horizon of the black hole. $S_{BH}$ is corrected semiclassical Bekenstein–Hawking entropy. $S_{BH}^{\prime }$ is a type of the Bekenstein–Hawking entropy included logarithmic-corrected. Through $S_{bh}$, $S_{BH}$, and $S_{BH}^{\prime }$, our understanding of the entropy of the black hole has deepened. Equation (32) represents a new expression for the quantum tunneling rate of spin-1/2 fermions at the event horizon $r_{H}$ of the Kiselev black hole, where $T_{H}$ denotes the Hawking temperature at the black hole horizon. Given that ${\left.\Delta \right|}_{r\to r_{H}}=0$, we derive the relation $r_{H}^{2}+a^{2}=2Mr_{H}+cr_{H}^{1-3\omega }$. The expressions for Γ, $T_{H}$, and $\omega_{0}$ demonstrate that these quantities are all dependent on $r_{H}^{2}+a^{2}$, which in turn depends on *c* and ω. Therefore, the quantum tunneling radiation of the Kiselev black hole surrounded by quintessence matter is characterized by distinctive features. Modifications such as quintessence matter, CFJ corrections, and aether-like field modifications each contribute to the quantum tunneling radiation of the black hole. The coefficient of the chiral correction term, denoted $b_{0}$, does not influence the quantum tunneling rate of the black hole, thus having no impact on the thermal quantum radiation described above.

## 3. Research on the Impact of Dark Energy and Lorentz-Breaking on Dirac Energy Levels in the Spacetime of the Kiselev Black Hole Surrounded by Quintessence

When examining quantum non-thermal radiation, the chiral correction term affects the energy level distribution of Dirac particles in the black hole spacetime, as well as the maximal crossing between the positive and negative energy levels of Dirac particles near the event horizon. To investigate the impact of dark energy and Lorentz-breaking on the Dirac energy levels in the curved spacetime of the black hole, we consider the equation governing the radial action *R* of Dirac particles in the region where $r>r_{H}$ within the curved spacetime, as shown below:

(51) $$\begin{array}{c} {\left(\Delta \frac{dR}{dr}\right)}^{2}=\frac{1-2a_{0}+2\lambda c_{t}^{2}}{1-2a_{0}+2\lambda c_{r}^{2}}\left\{{\left[E\left(r_{H}^{2}+a^{2}\right)\right]}^{2}-2anE\left(2Mr_{H}+cr_{H}^{1-3\omega }\right)+a^{2}n^{2}-\Delta \frac{\left(m^{2}-2b_{0}\gamma_{0}^{5}\right)r^{2}-d_{0}}{1-2a_{0}+2\lambda c_{r}^{2}}\right\} \end{array}$$

The left-hand side of this equation is a positive real number, thereby necessitating that the right-hand side satisfies the following conditions:

(52) $$\begin{array}{c} {\left[E^{2}\left(r_{H}^{2}+a^{2}\right)\right]}^{2}-2anE\left(2Mr_{H}+cr_{H}^{1-3\omega }\right)+a^{2}n^{2}-\Delta \frac{\left(m^{2}-2b_{0}\gamma_{0}^{5}\right)r^{2}-d_{0}}{1-2a_{0}+2\lambda c_{r}^{2}}\geq 0 \end{array}$$

By solving for the equality, we obtain the equation satisfied by the positive and negative energy levels of Dirac particles in the spacetime of the Kiselev black hole as follows:

(53) $$\begin{array}{c} {\left[E\left(r_{H}^{2}+a^{2}\right)-an\right]}^{2}=\Delta \frac{\left(m^{2}-2b_{0}\gamma_{0}^{5}\right)r^{2}-d_{0}}{1-2a_{0}+2\lambda c_{t}^{2}} \end{array}$$

From this equation, the positive and negative energy levels of Dirac particles in the spacetime of a Kiselev black hole are given by the following:

(54) $$\begin{array}{c} E_{\pm }=\frac{an}{r_{H}^{2}+a^{2}}\sqrt{\frac{\Delta }{{\left(r_{H}^{2}+a^{2}\right)}^{2}}\frac{\left(m^{2}-2b_{0}\gamma_{0}^{5}\right)r^{2}-d_{0}}{1-2a_{0}+2\lambda c_{r}^{2}}} \end{array}$$

It follows that the parameters $a_{0},\lambda ,b_{0},c,\omega$ all induce certain modifications to the distribution of positive and negative energy levels of the particles. From Equation (54), the energy level distribution in the spacetime of this black hole is given by

(55) $$\begin{array}{cc} \; & E>E_{+}\\ \; & E<E_{-} \end{array}$$

As $r\to r_{H}$, Equation (55) reveals that

(56) $$\begin{array}{c} \lim_{r\to r_{H}}E_{\pm }=\frac{an\left(2Mr_{H}+cr_{H}^{1-3\omega }\right)}{{\left(r_{H}^{2}+a^{2}\right)}^{2}}=\frac{an}{r_{H}^{2}+a^{2}}=E_{0} \end{array}$$

where $E_{0}$ represents the maximum value of the crossover energy levels $E_{+}$ and $E_{-}$. Under these conditions, the maximum energy condition for the quantum non-thermal radiation particles is $E\leq E_{0}$. Considering the limit r→∞, Equation (50) reveals that

(57) $$\begin{array}{c} {\left.E\right|}_{r\to \infty }∼\pm m \end{array}$$

where *m* denotes the mass of the spin-1/2 fermion. By combining Equations (56) and (57), the energy range of quantum non-thermal radiation at the black hole’s event horizon $r_{H}$ is given by

(58) $$\begin{array}{c} -m<E\leq E_{0} \end{array}$$

From Equations (8), (9) and (55)–(58), it can be seen that the distribution of fermionic levels in the spacetime of this black hole surrounded by dark energy is related to the dark energy component ω. This is a significant difference from other stationary black holes. Equation (58) can be considered as a new expression for the energy range of quantum non-thermal radiation particles from stationary black holes. The physical significance of this expression is as follows: particles exhibit wave-particle duality, and radiation particles can exist in both positive and negative energy states. There is a forbidden zone between these energy states, where no particles can exist. When the energy *E* in the negative energy state satisfies the condition in Equation (58), a phenomenon similar to the Klein mechanism occurs, where particle pairs are generated through quantum tunneling, resulting in radiation particles. This effect is known as quantum non-thermal radiation. In fact, this is a form of stimulated radiation of the black hole. When a black hole undergoes stimulated radiation, spontaneous radiation is also inevitably present, which corresponds to the Starobinsky–Unruh process. Both stimulated and spontaneous radiation are independent of the black hole’s temperature, and the radiative quantum non-thermal effects cause a reduction in the black hole’s rotation and angular momentum.

## 4. Discussion

The quantum tunneling rate $\Gamma^{\prime }$, Hawking temperature $T_{H}^{\prime }$, and Bekenstein–Hawking entropy $S_{BH}^{\prime }$ of this black hole after Lorentz-breaking and quantum perturbative corrections are given by Equations (40), (41) and (43), respectively.

Equations (32) and (33) are the corresponding results in semiclassical theory. By denoting the uncorrected Hawking temperature and tunneling rate as $T_{0}$ and $\Gamma_{0}$, respectively, the ratio of the tunneling rates before and after the correction can be obtained as follows:

(59) $$\begin{array}{c} \frac{\Gamma_{0}}{\Gamma }=\exp\left\{\left(-\frac{E-E_{0}}{T_{0}}\right)\left[1-{\left(\frac{1-2a_{0}+2\lambda c_{t}^{2}}{1-2a_{0}+2\lambda c_{r}^{2}}\right)}^{1/2}\right]\right\} \end{array}$$

Equations (40) and (41) represent the corresponding results including *ℏ* perturbation corrections. Therefore,

(60) $$\begin{array}{c} \frac{\Gamma_{0}}{\Gamma^{\prime }}=\exp\left\{\left(-\frac{E-E_{0}}{T_{0}}\right)\left[1-{\left(\frac{1-2a_{0}+2\lambda c_{t}^{2}}{1-2a_{0}+2\lambda c_{r}^{2}}\right)}^{1/2}\left(1+\sum_{i=1}\hbar^{i}\alpha_{i}^{\prime }\right)\right]\right\}. \end{array}$$

Equations (59) and (60) show the effects of Lorentz-breaking corrections and *ℏ* perturbation corrections on the quantum tunneling rate of this black hole. Since $T_{H}\left(T_{H}^{\prime }\right)$ is related to the dark energy parameter ω, and $T_{0}$, Γ, $\Gamma^{\prime }$, and the Bekenstein–Hawking entropy are all related to the Hawking temperature of the black hole, it is necessary to further explain the effect of the dark energy parameter ω on the black hole temperature and the Bekenstein–Hawking entropy. Using $T_{H}=\frac{2r_{H}-2M+c\left(1-3\omega \right)r_{H}^{1-3\omega }}{4\pi (r_{H}^{2}+a^{2})}{\left(\frac{1-2a_{0}+2\lambda c_{t}^{2}}{1-2a_{0}+2\lambda c_{r}^{2}}\right)}^{1/2}=T_{0}{\left(\frac{1-2a_{0}+2\lambda c_{t}^{2}}{1-2a_{0}+2\lambda c_{r}^{2}}\right)}^{1/2}$ and $S_{BH}=S_{bh}\left(\frac{1-2a_{0}+2\lambda c_{t}^{2}}{1-2a_{0}+2\lambda c_{r}^{2}}\right)$ as an example, the dependence of the temperature and the entropy and other physical quantities on ω is shown in the form of a graph.

From this table, it can be seen that when ω takes values in the range $-1<\omega <-\frac{1}{3}$, as ω increases, both $T_{0}$ and $T_{H}$ decrease. This reflects the influence of dark energy on the Hawking temperature of this black hole. Considering the Lorentz-breaking correction effects, the same value of ω corresponds to different $T_{0}$ and $T_{H}$, with $T_{H}<T_{0}$, which indicates that the Lorentz-breaking effect modifies $T_{0}$. This is what distinguishes this black hole from other types of black holes and makes it unique. From Table 1, it can be seen that ω has a certain effect on $S_{bh}$. $S_{BH}$ is influenced not only by ω but also by the Lorentz-breaking correction parameter. As ω increases, both $S_{bh}$ and $S_{BH}$ decrease.

We can use the Stefan–Boltzmann law to estimate the radiation rate and lifetime of this black hole. This helps illustrate the thermodynamic evolution of the black hole. According to the Stefan–Boltzmann law, the radiation rate of this black hole is as follows:

(61) $$\begin{array}{c} \frac{dE^{\prime }}{dt}=\sigma^{\prime }A_{K}{\left(T_{H}^{\prime }\right)}^{4} \end{array}$$

where $\sigma^{\prime }$ is the Stefan–Boltzmann constant. $A_{k}$ is given by Equation (48), and $T_{H}^{\prime }$ is given by Equation (41). Clearly, *M*, ω, $a_{0}$, $c_{r}$, $c_{t}$, and $\hbar^{i}$ all influence $\frac{dE^{\prime }}{dt}$. $E^{\prime }$ is the total energy of this black hole. By substituting Equations (41) and (33) into Equation (61), we obtain the following:

(62) $$\begin{array}{cc} \frac{dE^{\prime }}{dt} & ={\left(\frac{dE^{\prime }}{dt}\right)}_{L}{\left(1+\sum_{i=1}\hbar^{i}\alpha_{i}/S_{BH}\right)}^{-4}\\ \; & ={\left(\frac{dE^{\prime }}{dt}\right)}_{o}{\left(\frac{1-2a_{0}+2\lambda c_{r}^{2}}{1-2a_{0}+2\lambda c_{t}^{2}}\right)}^{-2}{\left(1+\sum_{i=1}\hbar^{i}\alpha_{i}/S_{BH}\right)}^{-4} \end{array}$$

where ${\left(\frac{dE^{\prime }}{dt}\right)}_{L}$ is the radiation rate that includes the Lorentz-breaking corrections. ${\left(\frac{dE^{\prime }}{dt}\right)}_{o}$ is the original radiation rate without corrections. From Equation (62), it can be seen that Lorentz-breaking and $\hbar^{i}$ perturbative corrections have certain effects on the thermodynamic evolution of this black hole. When the mass of the black hole reduces to the minimum value $M_{\min}$, the black hole will no longer radiate particles and will leave a residual mass $M_{\mathrm{res}}=M_{\min}$ [37]. We can estimate the evolution lifetime of this black hole. In Equations (61) and (62), $\sigma^{\prime }=\frac{2\pi^{5}K_{B}^{4}}{15\hbar^{3}c^{2}}$ and $A_{k}∼16\pi G^{2}c^{-4}M^{2}$, therefore, the radiation rate of this black hole is estimated as follows:

(63) $$\begin{array}{c} \frac{dE^{\prime }}{dt}∼{\left(\frac{dE^{\prime }}{dt}\right)}_{o}\Gamma^{\prime }∼{\left(\frac{dE^{\prime }}{dt}\right)}_{o}\Gamma \left(erg/s\right) \end{array}$$

where *M* is in grams. The tunneling rates $\Gamma^{\prime }$ and Γ are both estimated to be 1. It can be calculated that the radiation rate of this black hole is approximately:

(64) $$\begin{array}{c} \frac{dE^{\prime }}{dt}∼10^{46}\left(M^{-2}\right)\left(erg/s\right) \end{array}$$

Therefore, the lifetime of this black hole is estimated as $t∼10^{-27}M^{3}S∼10^{67}{\left(\frac{M}{M_{⊙}}\right)}^{3}$ (year). This shows that the lifetime of the black hole is directly related to its mass. The larger the mass *M*, the longer the lifetime *t*, and vice versa.The main content of the above research is the thermodynamic evolution characteristics of this black hole under the modifications of Lorentz-breaking and *ℏ* perturbation. Based on the modifications of the Hawking temperature and Bekenstein–Hawking entropy under Lorentz-breaking and *ℏ* perturbation theory, it can be seen that the specific heat capacity of this black hole, $C=T_{H}^{\prime }\left(\frac{𝜕S_{BH}^{\prime }}{𝜕T_{H}^{\prime }}\right)$, is also modified. These modifications are all meaningful research results. Since the dynamical Equation (18) for spin-1/2 fermions with Lorentz-breaking corrections is a semiclassical equation, the quantum tunneling rate, Hawking temperature and Bekenstein–Hawking entropy of the black hole obtained above are all results within the semiclassical theory, and these results are related to ω. It should be noted that the range of ω considered in the above study is $-1<\omega <-\frac{1}{3}$. When ω=0, it corresponds to the dust case, while $\omega =-\frac{1}{3}$ represents the radiation case. ω=−1 corresponds to the cosmological constant model, and $\omega =-\frac{1}{3}$ is associated with the so-called $R_{h}$ universe model [38,39]. Additionally, it is important to emphasize that the results presented above are derived within the framework of semiclassical approximation theory. To incorporate the quantum corrections due to the Planck constant *ℏ*, the term $\hbar \Omega_{u}$ should be retained in Equation (18). This modification enables the study of perturbative effects of *ℏ* based on the semiclassical Equation (18). The classical components of $R_{\pm }$ and *E* can be expressed as a series expansion in powers of *ℏ*, yielding expressions for quantum tunneling, Hawking temperature, and Bekenstein–Hawking entropy with higher-order corrections in *ℏ*. Furthermore, it is essential to highlight that all types of black holes exhibit quantum thermal effects, which are linked to the Hawking temperature. These effects are present in both stationary and non-stationary black holes, which exhibit both quantum thermal effects and quantum non-thermal effects. Static black holes, however, do not display quantum non-thermal effects. It should be further explained that we chose Equation (19) and defined $u_{\mu }$ instead of $u^{\mu }$. Our choice can simplify the calculation. The above research methods and conclusions are meaningful for the study of dynamical corrections of fermions with arbitrary spins and the tunneling of other types of black holes. The methods employed in this study are not applicable to bosons, and the relevant theory for scalar fields should be used for further investigation. It should be further noted that the above research methods can be applied to study quantum tunneling radiation specific characteristics of other fermions.

## Figures and Tables

**Table 1:** The effect of the dark energy parameter ω on the black hole temperature.

ω	−0.85	−0.8	−0.75	−0.7	−0.6	−0.5	−0.4	−0.37	−0.34
$T_{0}$	0.908426	0.675019	0.510699	0.393292	0.245706	0.164148	0.116817	0.106711	0.0979722
$T_{H}$	0.900012	0.668767	0.505969	0.389649	0.24343	0.162628	0.115735	0.105722	0.0970647
$S_{bh}$	169.612	124.936	94.8502	74.1562	49.2101	35.9299	28.3088	26.673	25.2503
$S_{BH}$	166.484	122.632	93.1013	72.7889	48.3027	35.2674	27.7869	26.1812	24.7847

M=1, a=0.6, c=0.4, $a_{0}=0.1$, λ=0.11, $c_{t}=0.3$, $c_{r}=0.4$.

## Data Availability

Data is contained within the article.
